# Ten years of marketing approvals of anticancer drugs in Europe: regulatory policy and guidance documents need to find a balance between different pressures

**DOI:** 10.1038/sj.bjc.6602750

**Published:** 2005-08-23

**Authors:** G Apolone, R Joppi, V Bertele', S Garattini

**Affiliations:** 1 Istituto di Ricerche Farmacologiche Mario Negri, Milan, Italy; 2 Dipartimento Farmaccutico, Unità Sanitaria Locale di Verona, Verona, Italy

**Keywords:** oncology drugs, marketing approval, regulatory, end points, EMEA, FDA

## Abstract

Despite important progress in understanding the molecular factors underlying the development of cancer and the improvement in response rates with new drugs, long-term survival is still disappointing for most common solid tumours. This might be because very little of the modest gain for patients is the result of the new compounds discovered and marketed recently. An assessment of the regulatory agencies' performance may suggest improvements. The present analysis summarizes and evaluates the type of studies and end points used by the EMEA to approve new anticancer drugs, and discusses the application of current regulations. This report is based on the information available on the EMEA web site. We identified current regulatory requirements for anticancer drugs promulgated by the agency and retrieved them in the relevant directory; information about empirical evidence supporting the approval of drugs for solid cancers through the centralised procedure were retrieved from the European Public Assessment Report (EPAR). We surveyed documents for drug applications and later extensions from January 1995, when EMEA was set up, to December 2004. We identified 14 anticancer drugs for 27 different indications (14 new applications and 13 extensions). Overall, 48 clinical studies were used as the basis for approval; randomised comparative (clinical) trial (RCT) and Response Rate were the study design and end points most frequently adopted (respectively, 25 out of 48 and 30 out of 48). In 13 cases, the EPAR explicitly reported differences between arms in terms of survival: the range was 0–3.7 months, and the mean and median differences were 1.5 and 1.2 months. The majority of studies (13 out of 27, 48%) involved the evaluation of complete and/or partial tumour responses, with regard to the end points supporting the 27 indications. Despite the recommendations of the current EMEA guidance documents, new anticancer agents are still often approved on the basis of small single arm trials that do not allow any assessment of an ‘acceptable and extensively documented toxicity profile’ and of end points such as response rate, time to progression or progression-free survival which at best can be considered indicators of anticancer activity and are not ‘justified surrogate markers for clinical benefit’. Anticipating an earlier than ideal point along the drug approval path and the use of not fully validated surrogate end points in nonrandomised trials looks like a dangerous shortcut that might jeopardise consumers' health, leading to unsafe and ineffective drugs being marketed and prescribed. The present Note for Guidance for new anticancer agents needs revising. Drugs must be rapidly released for patients who need them but not be at the expense of adequate knowledge about the real benefit of the drugs.

Mankind has never had so much knowledge and understanding of cancer as at present and this has generated a large number of approaches for developing new anticancer agents. However, despite important progress in understanding the molecular factors underlying the development of cancer and the improvement in response rates with new drugs, long-term survival is still disappointing for most common solid tumours (Berrino *et al*, 1999; Coleman, 1999; Levi *et al*, 2000). Most of the improvement in terms of age-standardised mortality in the EU is probably attributable to primary and secondary prevention, but survival gains with pharmacological treatments for common advanced/metastatic cancers are still measured in months, not years.

Several factors explain this gap between the explosion of information from basic (preclinical) research and its relatively poor yield for patients (Lee *et al*, 2000; Bast *et al*, 2001; Nathan, 2002; Apolone, 2003; Lenfant, 2003; Crowley *et al*, 2004). One simple explanation is that very little of the modest gain is actually the result of new compounds discovered and marketed recently. Doubts have indeed been raised about the added value of the new ‘targeted’ molecules compared to the classic and less-expensive anticancer drugs (Garattini and Bertele', 2002; Leaf, 2003), also in the framework of drug approvals (Roberts and Chabner, 2004).

Although much of the development of anticancer drugs continues after the regulatory agencies' approval, the US and EU agencies, FDA and EMEA, play a major role in improving public health as they fall between clinical trials and (public) health care and are thus responsible for the first scientific evaluation of the quality, safety and efficacy of new drugs. A look at the regulatory agencies' performance suggests some improvements. The FDA itself has recently reported its experience with oncology drugs (Johnson *et al*, 2003; Dagher *et al*, 2004).

The present analysis summarises and evaluates the types of studies and end points used by the EMEA over the last 10 years to approve new anticancer drugs through the centralised procedure, and discusses the application of the current regulations.

## MATERIAL AND METHODS

This report is based on the information available on the EMEA web site. Current regulatory requirements for anticancer drugs promulgated by the Agency (Note for Guidance and other documents) were identified and retrieved in the relevant directory (http://www.emea.eu.int/). Information about empirical evidence supporting the approval of anticancer drugs was retrieved from the European Public Assessment Report (EPAR) (http://www.emea.eu.int/index/indexh1.htm), a list of documents available in the public domain that describes the steps, reasons, scientific summary, technical documents and commitment for approval of a given drug, and the summary of product characteristics. Documents were surveyed for new applications and later extensions from January 1995, when EMEA was set up, to December 2004. This evaluation only includes anticancer drugs for solid cancers approved by the EMEA through the centralised procedure, including hormone treatments but excluding drugs for haematological cancers, such as leukaemia, lymphomas and multiple myeloma, and supportive therapies such as bisphosphonates.

We collected the following information: name of the active substance, year of approval, indication, design of the pivotal trials, number of patients in each trial, primary and secondary end points supporting the approval and difference in survival between arms, when available. We used the following definitions: a new indication was the first approval for a given drug, and later approvals were considered extensions; the trial design was classified as a randomised comparative (clinical) trial (RCT), or randomised noncomparative (phase II) trial (RNCT) or a single-arm trial (SAT) or other; the number of patients was generally the number included in the trial; the primary efficacy end points were those reported in the EPAR and were classified as survival, time to progression/progression-free survival (TTP/PFS) or response rate.

## RESULTS

### Current regulatory guidance for anticancer drugs

According to the guideline documents, phase III randomised comparative trials are generally required for marketing authorisation. The minimum requirement is normally one controlled trial with statistically compelling and clinically relevant results. As there is general demand for replication of scientific results, it is usually recommended to plan more than one study in the phase III programme. Directive 75/318/EEC provides for the possibility of granting marketing authorisation under exceptional circumstances, when in the present state of scientific knowledge, comprehensive information on the efficacy and safety under normal conditions of use cannot be provided.

In this situation, should an applicant consider that phase II studies have unequivocally established outstanding benefits of the new agent for the target patients, the applicant must fully justify this position before authorisation can be granted. In addition, a further trial programme must be agreed, together with any other experimental studies deemed necessary on the basis of adverse drug reactions. The CPMP Note for Guidance ‘Evaluation of anticancer medicinal products in man’ (CPMP/EWP/205/95 rev.2, 19 September 2002) presents guidelines on the requirements for authorisation for all anticancer drugs, particularly cytotoxic/cytostatic agents, and is intended to assist applicants in regular and exceptional circumstances (http://www.emea.eu.int/). It defines phase III trials as (therapeutic confirmatory) disease-oriented trials that seek to confirm the activity of the product seen in phase II (therapeutic exploratory) studies in the claimed indication, and specifies that a phase III clinical trial is comparative in nature and should allow a full evaluation of the active agent selected at the end of phase II. When the comparator is established, noninferiority trials are possible. Where this is not the case, a superiority trial is generally required.

Appropriate efficacy end points include progression-free/recurrence-free/relapse-free survival, overall survival, response rate and symptom control/quality of life. The primary end point should be selected *a priori* and justified on clinical relevance and methodological considerations. Randomised comparative (clinical) trial are normally always required, noncomparative studies being considered acceptable only in the case of pretreated patients when no established regimens do exist. This applies in very specific circumstances that are summarised in Box 1.

### Data supporting approval of anticancer drugs

We identified 14 anticancer drugs for 27 different indications (14 new applications and 13 extensions). Table 1 shows the main characteristics of each drug approved from 1995 to 2004, according to the indications. In general, a drug was approved on the basis of results from phase II or phase III studies. In one case only, approval was granted without empirical data, on the basis of a bibliographic review of nonclinical and clinical data (mitotane, for adrenal cortical carcinoma).

Often a drug was first approved on the basis of preliminary evidence from RNCT. Subsequently, new data from comparative studies made it possible to define the initial indication better or to extend the use of the drug to other diseases. For example, docetaxel was approved in 1995 under exceptional circumstances as single agent for the treatment of advanced/metastatic breast cancer, after failure of cytotoxic therapy that included an anthracycline or an alkylating agent. No data from phase III RCTs were submitted. In 1997, data from two RCTs were available, and the indication was confirmed and extended to two other clinical situations in advanced breast cancer patients. Eventually, in 1999, docetaxel was also approved for locally advanced or metastatic non-small-cell lung cancer on the basis of several RCTs. All together, docetaxel has five different indications, three in specific clinical situations for advanced breast cancer and two for advanced or metastatic non-small-cell lung cancer.

Overall, 48 clinical trials were used for approval of the 27 indications; RCT and response rate were the study design and the end points most frequently adopted (25 out of 48, and 30 out of 48). In 13 cases, the EPAR explicitly reported the differences between arms in terms of survival (reported as primary or secondary end points): the range was 0–3.7 months, and the mean and median differences were 1.5 and 1.2 months (Table 2). The mean numbers of patients recruited were, respectively 92, 222 and 406 for SAT, RNCT and RCT.

Table 3 shows the study design and the type of primary end point of the main studies supporting the 27 indications: phase III RCTs were available for half the indications, followed by SAT for 30% and RNCT for 14%; approval was based on at least two RCTs only for five products. As regard the end points supporting the 27 indications, the majority of cases (13 out of 27, 48%) involved evaluation of complete and/or partial tumour responses (Table 4). Time to TTP or PFS was next, while overall survival was available for only two indications There was no substantial difference in the trial design and end points between the first approval and the extension (data not reported).

## DISCUSSION

Despite the recommendations in the current EMEA guidance documents, the approval of new anticancer agents is often still based on small SAT that do not allow the evaluation of an ‘acceptable and extensively documented toxicity profile’, or on end points such as response rate, TTP or PFS which at best can be considered indicators of anticancer activity and not ‘justified surrogate markers for clinical benefit’. In only one case at least two RCT were available, and tumour shrinkage did not translate most of the time into significant survival benefit, usually reported as a secondary end point. In only one case, according to the information reported in the EPAR for this series of cases, approval was formally granted under the ‘exceptional circumstances’ condition.

It is beyond the scope of this paper to discuss the degree of innovation for new drugs and/or to make a cost analysis, as it has already been pointed out that the approval of most new anticancer drugs by EMEA offers no substantial advantages and puts further burdens on national health services (Garattini and Bertele', 2002). The present findings raise two issues: the general framework of anticancer drug approval, regulatory tools, recommendations, directives, etc. and specific ways to improve the development and evaluation of these drugs (clinical trial design).

The FDA recently reported that end points other than survival were the basis for approval of most (68%) oncology drug marketing applications following regular approval, and for all the applications following the accelerated approval procedure. In addition, 25 out of 71 (35%) drugs were granted an application without an RCT (Johnson *et al*, 2003; Dagher *et al*, 2004). The EMEA, established in 1995, has less formal regulatory tools to differentiate between regular and special/accelerated approval, and specific situations are treated case by case. In general, a distinction is made for anticancer drugs when no established treatment exists: in this case, an SAT may be the basis for approval. EMEA recently published a report on its 10-year experience in oncology (Chapelin *et al*, 2004) evaluating the study design and end points used; although details are not yet available, findings are very similar to those reported in this paper for solid tumours.

Despite these formal differences between agencies, the output is similar. Pharmaceutical companies, under the pressure of investors, test their new molecules on human beings at the earliest possible point in order to obtain quick approval, without fully knowing the true mechanism by which these new drugs exert their clinical action. Often strange and specific subsamples of progressing or refractory patients are selected to obtain the status of ‘accelerated approval’ or ‘under exceptional circumstances’, using the simplest possible study design. This means that most candidate anticancer drugs are eventually marketed on the basis of little firm evidence. The tendency to anticipate an earlier than ideal point along the drug approval path, and the use of not fully validated surrogate end points in nonrandomised studies can be considered either an important step towards a new way of protecting public health by ensuring that beneficial drugs are made available as quickly as possible, or a dangerous shortcut that might jeopardise consumers' health, allowing unsafe and ineffective drugs to be marketed and prescribed (Castro, 2002; Horton, 2004; Psaty *et al*, 2004).

Although pharmacological treatment is only part of the care of cancer patients, the use of drugs in each stage of the disease (neoadjuvant, adjuvant, advanced, metastatic and terminal) is the hub of recommended approaches, and a lack of evidence about their real benefit/risk ratio may lead to overestimation of the role of drugs in the cure or control of the disease and consequently to overtreatment, high costs and poor outcomes. On the other hand, though it is generally accepted that a complete dossier would be desirable, sometimes confirmative phase III studies are hard to arrange for ethical or feasibility reasons and most patients with advanced disease cannot wait for the full approval, by which time they would probably be dead. This dilemma may be partially solved by involving patients' organisations so cancer patients are represented in the EMEA evaluation process to help identify fast-track designation and by starting information and communication initiatives at each national level to prepare patients to take the risk of a partially proven therapy.

In addition to what has already been suggested to improve the policy of regulatory systems, in both the USA (Leaf, 2003; Roberts and Chabner, 2004) and EU (McVie, 2002; Garattini and Bertele', 2002; Redmond, 2004), current guidance documents (Note for Guidance, NfG) need to be modified in order to balance the pharmaceutical companies' aims with the public health systems' mission, also in the light of the methodological challenges introduced by new, molecularly targeted agents.

Phase II studies are vital in the development of new drugs. A first way to improve phase II is to require randomisation of patients in order to obtain more reliability results. There is no reason why this cannot be done when phase I has determined the MTD, beginning with the first patient to enter phase II. Patients who are not treated with the drug under investigation could receive the best available care or the treatment they would have been given if the experimental drug had not been available. Since phase II patients have advanced disease, already treated with all the available options, but are usually not terminal, they were very likely to receive some other treatment, even an off-label one. The presence of a control group, not for comparative purposes, could establish whether there is actually any outstanding (unexpected) activity and help to plan a more rational phase III trial.

A critical aspect is the end point chosen in phase II and here the present NfG are rather vague. Ideally, it would be important to establish that the new drug to be considered clinically significant meaningfully increases survival. Objective responses – morphological or biochemical when they are significant – do not always correlate with clinical improvement. Similarly, disease stabilisation or PFS are subject to extensive bias, and it is very difficult to accept any PFS as real if it is not supported by an increase in overall survival.

No drugs in Europe received marketing approval by EMEA on the basis of a formal assessment of symptom control or of quality of life; improvement of these parameters should be more acceptable than objective tumour response, if the aim of treatment is palliative.

In any case, unless the new drug dramatically changes the natural history of a specific tumour, phase III trials should be compulsory for several reasons. First of all, there is convincing, and in some cases conclusive evidence that the classification of solid tumours masks heterogeneous components, causing considerable difference in the prognosis, in terms of survival and response to treatment (Betensky *et al*, 2002). Even spectacular results obtained in some phase II cases may be related to a casual mix of patients with favourable gene profiles that are not shared by all patients with the same tumour. The recent results with *gefitinib* in lung cancer patients with mutations in the EGFR gene are striking but only a minority of the lung cancer population may have this mutation (Lynch *et al*, 2004; Paez *et al*, 2004).

Secondly, approving a drug with phase II data gives it a status of ‘active comparator’ in other studies, even if its efficacy has not been fully established. Thirdly, although the NfG stipulate that drugs with only phase II studies must undergo further trials, it is difficult to arrange phase III trials when the efficacy of an authorised drug is considered well established. It is not infrequent to hear ethical appeals in order to avoid further trials. Fourth, under the current legislation, it is extremely difficult to remove drugs from the market. Therefore, there is a high risk of drugs approved only with phase II trials being utilised in a number of patients before confirmative trials are carried out. Last but not least, ethical considerations are difficult to reconcile with studies whose aim is essentially commercial.

For these reasons it is suggested that new anticancer drugs be approved on the basis of phase II studies only in exceptional cases, when there is really outstanding, unprecedented or unexpected activity, and that therefore phase III comparative trials should generally be required.

The current NfG is acceptable for phase III trials except that they should be not only ‘confirmatory’ but also ‘comparative’. The type of comparison is also important. The noninferiority design is hard to accept for at least two reasons: first, anticancer agents are generally not very effective and therefore it is difficult to make sure that in a noninferiority trial without a placebo the standard drug actually shows efficacy; second, this doubt is reinforced by the limit for noninferiority which is usually relatively generous (15–20%) and therefore easy to achieve.

Again the preferred end point should be overall survival. It can be argued that for most tumours a median increase in survival of 1.2 months or so, even if statistically significant, is of questionably utility because it is so small and there are always many potential confounding factors. It is evident that a ‘positive risk-benefit’ assessment is difficult in the absence of information about the patients’ quality of life. If other parameters are used, such as measures of clinical/therapeutic benefit, they should be supported by adequate studies to identify a real improvement in quality of life through validated psychometric and pragmatic scales (Chassany *et al*, 2002).

Phase III and phase II studies should be required to include large proportion of elderly people and, when appropriate, pediatric trials should be done.

The new NfG should give proper importance to the emerging differences in outcome due to different genotype profiles (Roberts and Chabner, 2004). For patients responding well to a treatment (usually a small proportion) retrospective, preplanned analyses should be done to see if there is a genotype that identifies responders or nonresponders. This information would be useful for granting an indication that takes account of the characteristics of likely responders, avoiding a broad indication under which many patients will be treated with no advantage. It is clear that such studies are unlikely to interest industry because they will shrink the market.

An example of an approval based on molecular requirements is trastuzumab for advanced breast cancer patients positive for HER-2 (Vogel *et al*, 2002), where confirmative phase III studies are still running. In the meantime, how many patients have been treated, at a high cost for society, without knowing the real level of efficacy?

This paper has several limitations. First of all, the evaluation only pertains to anticancer drugs approved by EMEA following the centralised procedure and it must be borne in mind that some important anticancer drugs such as gemcitabine were approved in Europe during the study period using alternative regulatory procedures. Secondly, an analysis of the group of agents that were not approved by EMEA or voluntarily withdrawn by a pharmaceutical company would allow a more complete assessment; unfortunately, this information is not available as it is confidential and there is no means of tracing any public information on procedures stopped early. Thirdly, the evaluation is based only on information for drugs for solid tumours and the findings and comments cannot be extended to drugs for other cancers. Fourth, individual drugs have gained their indications with a specific path in some very restricted forms of cancer and a qualitative discussion of each example might help to identify critical points in the development and evaluation process and clarify potential regulatory and clinical implications; such discussion is beyond the aims of this paper, but this kind of evaluation is available elsewhere for some of the drugs assessed (Pignatti *et al*, 2002). Finally, the data used in this appraisal came from documents made available to the public by EPAR, such as the scientific summary; in general, such documents are not standardised and differ in length, completeness, level of discussion and amount of technical detail reported, thus making it difficult to identify and retrieve the information required for the evaluation; the heterogeneity in structure, contents and organisation might have introduced a bias.

In conclusion, the present NfG for new anticancer agents need revising. There should be a very strong obligation for phase III trials unless we are lucky enough to have found a ‘magic bullet’, but these are very rare! The design of phase II studies should be improved to make sure that a positive result points to a real need for phase III studies. In the light of the EMEA's attitude, it is equally important that the NfG are not only revised but also strictly applied. Of course drugs should be rapidly released for patients who need them but not at the expense of adequate knowledge about the real benefit. The pharmaceutical industry's urgency to obtain a slice of a flourishing market should be balanced by the need to provide drugs that meet real need without posing an undue burden on European national health services.

## Figures and Tables

**Box 1:**
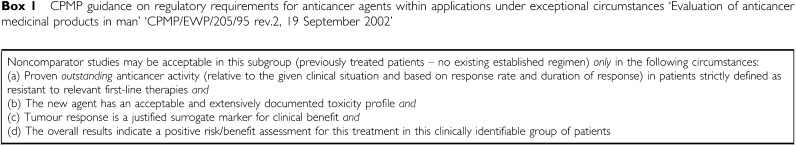
CPMP guidance on regulatory requirements for anticancer agents within applications under exceptional circumstances ‘Evaluation of anticancer medicinal products in man’ ‘CPMP/EWP/205/95 rev.2, 19 September 2002’

**Table 1:** Anticancer drugs approved by EMEA from January 1995 to December 2004

**Drug**	**Year**	**Indication**	**Trial design**	**No. of patients**	**Primary end points**
Docetaxel^a^	1995	ABC, second line, monotherapy	6 SAT	228	RR
Topotecan	1996	AOC, second line	RCT	235	RR
Toremifene	1996	ABC, first line, postmenopausal	3 RCT	648, 415, 463	RR, RR, RR
Doxorubicin pegylated^b^	1996	AIDS – Kaposi	SAT	247	RR
			SAT	137	RR
Docetaxel	1997	ABC, second line, with capecitabine	RCT	429	TTP
		ABC, first line, with doxorubicin	RCT	477	TTP
Temozolide	1998	GBM, refractory	RCT, SAT	225, 138	PFS, PFS
		Anaplastic astrocytoma, first line	SAT	162	PFS
Tasonermin	1999	Limbs sarcoma	4 SAT	39,23,23,103	RR, RR, RR, RR
Paclitaxel	1999	AIDS, Kaposi, second line	SAT	107	RR
Docetaxel	1999	A-NSCLC, second line	3 RCT	NA, NA, NA	OS, OS, OS
		A-NSCLC, first line with cis-platinum	RCT	1220	OS
Doxorubicin pegylated	2000	AOC, second line	RCT	474	TTP
Doxorubicin pegylated	2000	ABC, first line, when A nonindicated	2 RCT	509, 301	PFS, PFS
Paclitaxel	2000	ABC, second line or when A nonindicated	NC-RCT	312	TTP
Paclitaxel	2000	ABC, second line	NC-RCT	120	PFS
Trastuzumab	2000	ABC, first line in combination with paclitaxel, when A nonindicated	RCT	469	TTP
		ABC, second line, single agent	SAT	222	RR
Altretinoin	2000	AIDS-Kaposi, after antiretroviral therapy	RCT	238	RR
Capecitabine	2000	A-CRC	2 RCT	605, 602	RR, RR
Capecitabine	2000	ABC, second line with docetaxel	RCT	511	TTP
		ABC, second line, single agent when A nonindicated	SAT	135	RR
Fulvestrand	2001	ABC, postmenopausal, second line	2 RCT	541, 473	TTP, TTP
Imatinib	2002	Adult GIST	NC-RCT	117	RR
Cetuximab	2004	A-CRC, second line, in combination with irinotecan	NC-RCT, 2 SAT	329, 138, 57	RR, RR, RR
Mitotane	2004	Adrenal cortical carcinoma	Review	Review	—

ABC=advanced breast cancer; AOC=advanced ovarian cancer; GBM=gliobastoma multiforme; A-CRC=advanced colorectal cancer; A-NSCLC=advanced non-small-cell lung cancer; GIST=gastrointestinal stromal tumour; A=anthracycline; RCT=randomised clinical trial; SAT=single-arm trial; NC-RCT=noncomparative RCT; OS=overall survival; TTP=time to progression; PFS=progression-free survival; RR=response rate; NA=not available.

a Docetaxel was first approved under exceptional circumstances on the basis of six pooled SAT, for a total of 228 cases. Later, in 1997, full approval was granted on the basis of two RCT, with 326 and 392 patients and RR as supporting end point.

b Doxorubicin pegylated was first approved on the basis of SAT. Later, full approval was granted on the basis of two RCT, with 258 and 241 patients and with therapeutic response and response rate, respectively, as supporting end points.

**Table 2:** Evaluation of anticancer drugs by EMEA from 1995 to 2004: summary of the 48 studies used as basis for approval

**Clinical trial design (48 trials)**	**Type of end point (primary) (48 trials)**	**Difference in survival, when available (13 trials)**
RCT 25	Survival 4	Range 0–3.7 months
SAT 19	Resp. rate 30	Mean 1.5 (months)
NC-RCT 4	TTP/PFS 14	Median 1.2 (months)

RCT=randomised clinical trial; SAT=single-arm trial; NC-RCT=noncomparative RCT; TTP=time to progression; PFS=progression-free survival.

**Table 3:** Design of the main studies supporting 27^a^ indications

	**No.**	**%**
Phase III RCT	14	52
NC-RCT	4	14
SAT	8	30
Other^b^	1	4

RCT=randomised clinical trial; SAT=single-arm trial; NC-RCT=noncomparative RCT.

a In at least two cases (docetaxel and doxorubicin pegylated), further comparative RCT were carried out to support preliminary findings.

b Approval without empirical data, supported by bibliographic review of nonclinical and clinical data.

**Table 4:** Primary efficacy end points of the main studies supporting 27 indications

	**No.**	**%**
Overall survival	2	7
TTP/PFS	11	41
Response rate	13	48
Other^a^	1	4

TTP=time to progression; PFS=progression-free survival.

a Approval without empirical data, supported by bibliographic review of nonclinical and clinical data.
